# Building a research career in psychiatry: challenges and opportunities

**DOI:** 10.1192/j.eurpsy.2023.198

**Published:** 2023-07-19

**Authors:** S. Guloksuz

**Affiliations:** ^1^Psychiatry, Maastricht University, Maastricht, Netherlands; ^2^Psychiatry, Yale School of Medicine, New Haven, CT, United States

## Abstract

**Abstract:**

In this interactive session, we will attempt to create a roadmap toward a research career in psychiatry, particularly in challenging settings. Participants will be able to share their experiences and ask questions. Through dialectical discourse, we will identify the essential skills and key competencies for a research career and how to develop them through key strategies and practical exercises. We will try to answer these questions and more. “I like research, but I don’t know where to start?” “I know writing is important, but how can I make the process less painful and even perhaps enjoy writing?” “There is no research tradition in my workplace; how can I find a mentor?” “How can I build confidence in presenting at scientific conferences?” “It’s important to master hard skills, but what could we do with soft skills?”

**Disclosure of Interest:**

None Declared

